# Comparing subcutaneous tissue responses to freshly mixed and set root canal sealers

**Published:** 2009-10-10

**Authors:** Setareh Derakhshan, Alireza Adl, Masoud Parirokh, Fatemeh MashadiAbbas, Ali Akbar Haghdoost

**Affiliations:** 1 *Department of Endodontics, Kerman Oral and Dental Diseases Research Center, Dental School, Hamadan University of Medical Sciences, Hamadan, Iran*; 2 *Department of Endodontics, Dental School, Shiraz University of Medical Sciences, Shiraz, Iran*; 3 *Department of Endodontics, Dental School, Oral & Dental Diseases Research Center, Kerman University of Medical Sciences, Kerman, and Iranian Center for Endodontic Research, Tehran, Iran*; 4 *Department of Oral and Maxillofacial Pathology, Dental School, Shahid Beheshti Medical University, Tehran, Iran*; 5 *Department of Epidemiology and Biostatistics, Physiology Research Center, Kerman University of Medical Sciences, Kerman, Iran, Honorary lecturer in London School of Hygiene and Tropical Medicine, London, UK*

**Keywords:** AH26, AH Plus, Biocompatibility, Implantation, RoekoSeal, Root canal sealer, Subcutaneous

## Abstract

**INTRODUCTION:** The purpose of this study was to compare the subcutaneous tissue responses of freshly mixed or set endodontic root canal sealers (*i.e. *RoekoSeal, AH26, AH Plus) in Wistar Albino rats.

**MATERIALS AND METHODS:** Seventy-two male albino rats weighing 200-250g were used. The animals were randomly divided into six groups of 12 rats each. Root canal sealers were implanted in subcutaneous tissue in both freshly mixed and set conditions. The animals were sacrificed after 7, 14, and, 60 days. After histological preparation and Hematoxylin and Eosin (H&E) staining, the specimens were evaluated for capsule thickness, severity and extent of inflammation, and necrosis. Results were statistically analyzed using Multivariate ANOVA test.

**RESULTS:** Differences between set and freshly mixed root canal sealers were significant (P=0.014), but not significant between test materials and controls, except for capsule thickness and extent of inflammation between control and AH26 (P=0.019 and P=0.006 respectively). The interaction between the type of material and setting condition was significant for capsule thickness and severity of inflammation in AH26 specimens at 14 and 60 days (P=0.001).

**CONCLUSION:** Based on the results of this study assessing the biocompatibility, both set and freshly mixed states can be used. [Iranian Endodontic Journal 2009;4(4):152-7]

## INTRODUCTION

One of the aims of endodontic treatment is to obturate the root canal system with an impervious, biocompatible, and dimensionally stable filling material. Obturation with gutta-percha cones and sealer are now accepted as the most reliable method for filling the root canal system. An ideal root canal sealer should be non-toxic to the periapical tissues and induce bone formation between the core material and dentinal root canal wall. This will prevent leakage as well as encourage and enhance healing of periapical lesions (1).

Considerable efforts have been made to investigate and enhance the physical and biological properties of sealers (2-5). Although various kinds of materials like zinc oxide eugenol (ZOE), resin cements, glass-ionomer and polyketone compounds have been used as root canal sealer, the ideal root canal sealer has yet to be found. Unfortunately, developing sealers that provide both good physico-chemical characteristics and biological compatibility is difficult. Well-tolerated materials by tissues may have limited sealing capacity, and vice-versa.

It has been shown that sealers should have both good sealing ability and biocompatibility for clinical use (6) as endodontic filling materials are placed directly into vital tissues. The tissue response to these materials is important as it may influence the outcome of the endodontic treatment (1). Periapical tissue reactions after root canal treatment and/or obturation may be influenced by preexisting diseases, elimination of pulp tissue, cleaning and shaping of the root canal, bacterial infection, filling technique, and the chemical nature of the sealer (1). For more information about sealers and more appropriate selection, they should be tested on animal models (7).

RoekoSeal Automix (RSA) silicon-based sealer was developed several years ago. It consists of polydimethyl siloxane, silicon oil, paraffin-base oil, hexachloroplatinic acid (catalyst), and zirconium dioxide (radiopaque material) (RSA Brochure).

Some of the results of leakage studies on RSA are conflicting; however the materials and methods utilized are very variable.

De-Deus *et al.* showed that RSA root canal sealer had significantly less bacterial penetration in comparison with AH Plus sealer *in vitro* (8). In contrast, Pereira *et al.* study found no significant difference *in vivo.* In their study the dog's teeth were immersed in India ink after canal obturation and post space preparation (9). Also a recent leakage study found no significant difference between AH26 and RSA (10). Previous research studies have shown that RSA has less cytotoxicity than other root canal sealer such as AH plus (11-15).

In a recent study RSA have shown significantly less cytotoxicity than Epiphany root canal sealer (16).

Gencoglu *et* *al.* showed that RSA performed well when injected subcutaneous tissues of rats. However, they did not compare the response of subcutaneous implantation of RSA to any other root canal sealers (4).

The aim of this study was to compare the biocompatibility of freshly mixed and set conditions of RSA and compare them with two well known root canal sealers (AH Plus and AH26) in albino rats.

## MATERIALS AND METHODS

The research protocol was approved by the Research Ethics Committee of Kerman University of Medical Sciences and experiment was carried out in accordance with the European Economic Community’s directive 24 November 1986 (86/609/EEC).

All the recommended points by Institutional Animal Care and Use Committee were observed in all different stages of the project.

We used 72 male Wistar albino rats weighing 200-250 g. The animals were anaesthetized by intraperitoneal administration of 47.5 mg/kg Ketamine HCl (Alfasan, Woerden, the Netherlands) and 0.01 mg/kg Rompun 2% (Alfasan, Woerden, the Netherlands). In each animal 4 regions of the dorsal skin of right and left sides were shaved (two anterior sites anterior, two posterior sites). For maximum pain relief, 0.1 mL of lidocaine 2% with epinephrine 1/80000 (Daroupakhsh, Tehran, Iran) was used as local anesthetic at the sites of implantation. Areas were disinfected with Povidone Iodine 10%, a small incision approximately 12 mm long was then made with a No.15 blade (Carl Martin, Solingen, Germany). With a blunt dissecting instrument a pocket was made to a depth of 20 mm to implant the tubes in the subcutaneous tissue. The animals were randomly divided into 2 groups for set and freshly mixed materials. Each group was divided into 3 subgroups of twelve rats, each for one time interval. Each rat received three implants which contained one of the materials presented in Table 1, and an empty tube as a control.

For freshly mixed groups, materials were prepared following the manufacturer’s instructions. Ethylene dioxide sterilized polyethylene tubes with a single lumen of 7 mm long and internal diameter of 1.7 mm were filled RSA (Roeko, Langenau, Germany) or AH Plus (Dentsply, De Tray, Konstanz, Germany) or AH26 (Dentsply De Trey, Konstanz, Germany). For set groups, root canal sealers were mixed according to the manufacturers’ instructions. The mixed materials were placed into polyethylene tubes using a sterile plastic instrument. The polyethylene tubes contained freshly mixed sealer. They were kept in an incubator in aseptic conditions for 24 h at 37^°^C for initial setting. The skin was then sutured with silk suture material (Supa, Tehran, Iran).

 At 7, 14, and 60 days after implantation, the animals were sacrificed by an overdose of Ketamine HCl. The implantation area was re- shaved and the skin and underlying connective tissue containing the implant was excised as a block section and kept in 10% formalin for at least 48 hrs. After fixation, a section parallel to the long axis of the tube was made. The tissues were prepared for Hematoxylin and Eosin (H&E) staining. A blinded pathologist evaluated the specimens. Tissue reactions were evaluated at both ends of the tubes to assess the following histological features outlined below.

**Table 1:** Comparison results of different materials, setting condition and interactions, using Multivariate ANOVA analysis

Materials based on different inflammatory indicators	*P value*	*power*
Capsule Thickness^*^	0.019	0.76
Severity of Inflammation	0.060	0.61
Extent of Inflammation^**^	0.006	0.86
Necrosis	0.170	0.44
All factors	0.056	0.91
Set versus freshly mixed	0.014	0.85
Interaction between material and setting condition^***^	<0.001	0.99

^*^ *Only the difference between controls and others were statistically significant at 7 and 14 day intervals. *

^**^ *Only the difference between controls and AH26 was statistically significant*

^***^ *The interaction between setting condition and materials was significant and it means that the effect of setting in all materials was not similar.*

1- The thickness of connective tissue capsule. The extent of connective tissue formation around the tube in ×100 field of vision and were measured by micrometer.

2- The severity of inflammation which was defined by the concentration of inflammatory cells in/around connective tissue capsule in ×40 field of vision:

+1: Less than 25 cell counts

+2: 25<cell count<50

+3: 50<cell count<75

+4: Over 75 cell counts

3- The extent of inflammation defined by the expansion of inflammation in ×40 field of vision:

+1: Less than capsule thickness

+2: Full capsule thickness

+3: Beyond capsule thickness

4- Necrosis was also assessed under ×40 field of vision and noted as either present or absent.

To assess the effects of set condition, time of observation, type of material, and the interactions between these factors, we used an ANOVA test model, Tukey test as the post-hoc test. We also compared materials with each other. All of the analyses were performed in SPSS version 11.5. The significance level was set at P<0.05.

## RESULTS

Multivariate ANOVA analysis showed significant differences between set and freshly mixed groups (P=0.01). Also the setting of materials did not have any influence on necrosis index (Table 1).

According to Table 1, there was no significant difference between the test materials and controls, except for capsule thickness (P=0.019) at 7 and 14 days intervals. For the extent of inflammation, the difference between controls and AH26 was significant (P=0.006).

**Figure 1:**
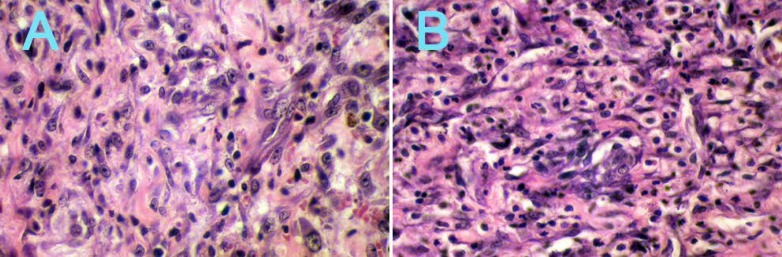
The severity of inflammation in set (A) and freshly mixed AH26 (B) after 14 days (×40)

The interaction between the type of the materials and the setting condition was significant for AH26 (*i.e.*, the setting condition did not have similar effect of the freshly mixed root canal sealers on the tissue reaction- P=0.001). Histological observation showed significant differences between set and freshly mixed AH26 root canal sealers in capsule thickness and severity of inflammation indexes (Figure 1) (P=0.001).

Capsule thickness in freshly mixed specimens of AH26 root canal sealer was significantly different at 7, 14, and 60 days intervals in comparison with set condition (P=0.001).

Severity of inflammation in freshly mixed specimens of AH26 was significantly different compared to set condition at 14 (Figure 1) and 60 day (P=0.001).

**Figure 2:**
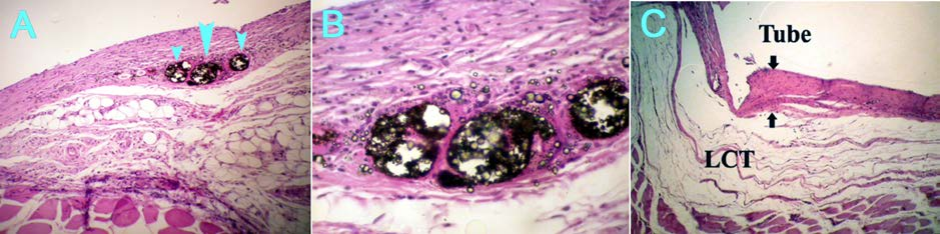
A) Freshly mixed AH plus after 60 days (×10) (Arrowheads: sealer extrusion) B) same figure (×40) C) freshly mixed RoekoSeal-Automixed after 60 days (×10) (Arrows: capsule thickness)

As time passed, all inflammatory indexes showed statistically significant decrease, which was observable in both set and freshly mixed groups (Figure 2), (P=0.01).

In all mentioned analyses (Table 1), statistical power was higher than 0.6, except for the effect of different materials on developing necrosis (power=0.44). Hence we can deduce that the calculated p values were reliable.

## DISCUSSION

There is some controversy regarding using freshly mixed or set states of root canal sealers for assessing tissue reaction to the materials in subcutaneous implantation studies. Some investigators believe that freshly mixed root canal sealers are flowable immediately after mixing; and therefore the tissue reaction may be influenced by the extrusion of root canal sealer to the surrounding subcutaneous areas. Therefore the set condition of the material would be preferred (2). In contrast, others have emphasized to use freshly mixed sealer as it is similar to clinical condition, where material may extrude to the periapical areas (5,7).

Traditional technique for evaluating biocompatibility of dental materials in *in vivo* studies is subcutaneous implantation in polyethylene or Teflon tubes (17-21). In this study, polyethylene tubes were used because of their inert nature which makes them a suitable test material for contact with living tissue.

In order to evaluate tissue reaction, Stanford suggested two time intervals (22). However, Olsson *et al.*, have suggested three time intervals (23). The present study implemented three time intervals: 7, 14, and 60 days.

In a research study, Huang *et al. *evaluated the cytotoxicity of set AH Plus and AH26 along with other set root canal sealers on human periodontal ligament (PDL) cells and a permanent hamster cell line (V79 cells). They found that the cytotoxic response of AH plus was less than AH26 (25). In the present study, AH plus showed tissue reaction during the early time intervals which was not significant in comparison with control specimens. Leonardo *et al.* *in vivo* study showed that the teeth filled with AH Plus resulted in better histological repair than other tested sealers (26).

In our study, capsule thickness over tested materials compared with controls showed significant difference (Table 1), though the capsule thickness compared within materials demonstrated no significant difference. Capsule formation rate was high particularly in freshly mixed materials in response to the foreign body reactions and toxicity of materials. In the 7 day specimens, thicker capsule could be observed; although in some specimens the severity of root canal toxicity prevented capsule formation. We assumed that early capsule formation is a sign of favorable biocompatibility of the root canal sealer, because inflammation is not severe enough to prevent fibroblasts forming a capsule. When a foreign body is not overtly irritating, it becomes either absorbed or encapsulated by the body.

It seems that capsule formation is an attempt to confine toxicity to a foreign body. Based on the toxicity comparing test materials at different time intervals, as time lapsed, all inflammatory indexes showed statistically significant decrease, which was observed in both set and freshly mixed groups (P=0.01).

In this study since the toxicity diminished after 60 days, lower capsule thickness became observable. A significant difference was found between AH26 and the controls when comparing the inflammation index. These severe reactions were especially obvious in 7 and 14 day intervals. In a biocompatibility study, using baboons teeth to evaluate tissue reaction to AH26, Pascon *et al.* concluded that at early observation periods (1-7 days), AH26 caused severe reactions, and at 2 and 3 yr observation periods, mild reactions were observed (27). In a further study, 14 days after subcutaneous implantation, AH26 showed moderate tissue reaction (28).

In this study we found a significant difference in capsule thickness between freshly mixed and set AH26 at all time intervals (P=0.001). Meanwhile, severity of inflammation was higher at 14 and 60 day intervals between freshly mixed and set AH26 (P=0.001). AH26 is a powerful irritant initially (23,29,30). According to Economides *et al.* the irritation of AH26 during the initial period can be attributed to the formaldehyde release as a decomposing product of hexamethylenetetramine. In long term studies, AH26 demonstrated good response in terms of tissue tolerance (30).

RSA is a silicon-based root canal sealer with good properties (31-35). Our study showed similar biocompatibility of RSA, AH Plus and AH26, not significantly different when they were implanted subcutaneously in set and freshly mixed condition. Biocompatibility of RSA showed that despite severe tissue reactions during 1-day intervals, at 30-day intervals, samples showed a well formed fibrous capsule around surrounding tissues (4). Lodiene *et al.* have shown that RSA in either fresh or set form has no cytotoxicity for 24 h (16). A study on periapical area of dog’s teeth have shown no significant difference between AH Plus and RSA in terms of dentin, cementum, and bone resorption, thickness of periodontal ligament and infiltration of inflammatory cells (36). In our study, RSA showed no significant difference to controls at all time intervals in both set and fresh status. Ørstaik and Mjör assessed set states (24 hours) to prevent sealer extrusion (2). Our study showed significant difference between capsule thickness and severity of inflammation in set and freshly mixed AH26 at 14 and 60 day intervals (P=0.001); the set condition showed a milder response. There was no significant difference between RSA and AH plus. Studies have shown the maximum toxicity of most sealers during the first 24 hours before setting; therefore the results must be interpreted with caution (2).

## CONCLUSION

RSA and AH Plus root canal sealers showed good biocompatibility. However, the extent of inflammation of AH26 was significantly higher than controls. AH26 root canal sealer also demonstrated significant difference between set and fresh states in certain time intervals.
